# Structure–Activity Relationships of the Tetrapeptide Ac-His-Arg-(*p*I)DPhe-Tic-NH_2_ at the Mouse Melanocortin Receptors: Modification at the (*p*I)DPhe Position Leads to mMC3R Versus mMC4R Selective Ligands

**DOI:** 10.3390/molecules24081463

**Published:** 2019-04-13

**Authors:** Katherine N. Schlasner, Mark D. Ericson, Skye R. Doering, Katie T. Freeman, Mary Weinrich, Carrie Haskell-Luevano

**Affiliations:** Department of Medicinal Chemistry & Institute for Translational Neuroscience, University of Minnesota, Minneapolis, MN 55455, USA; schlasner@gmail.com (K.N.S.); erics063@umn.edu (M.D.E.); skye.doering@gmail.com (S.R.D.); freem236@umn.edu (K.T.F.); maryweinrich@mail.usf.edu (M.W.)

**Keywords:** MC3R, MC4R, mixed pharmacology, tetrapeptides, melanocortins

## Abstract

The five melanocortin receptors (MC1R–MC5R) are involved in numerous biological pathways, including steroidogenesis, pigmentation, and food intake. In particular, MC3R and MC4R knockout mice suggest that the MC3R and MC4R regulate energy homeostasis in a non-redundant manner. While MC4R-selective agonists have been utilized as appetite modulating agents, the lack of MC3R-selective agonists has impeded progress in modulating this receptor in vivo. In this study, the (*p*I)DPhe position of the tetrapeptide Ac-His-Arg-(*p*I)DPhe-Tic-NH_2_ (an MC3R agonist/MC4R antagonist ligand) was investigated with a library of 12 compounds. The compounds in this library were found to have higher agonist efficacy and potency at the mouse (m) MC3R compared to the MC4R, indicating that the Arg-DPhe motif preferentially activates the mMC3R over the mMC4R. This observation may be used in the design of new MC3R-selective ligands, leading to novel probe and therapeutic lead compounds that will be useful for treating metabolic disorders.

## 1. Introduction

The melanocortin system consists of five receptors (MC1R–MC5R) [1,2,3,4,5,6,7,8] belonging to the class A family of G protein-coupled receptors (GPCRs). The melanocortin receptors are involved in numerous physiological functions and primarily signal through the G_αs_ pathway, increasing production of cyclic adenosine monophosphate (cAMP) upon receptor activation [9]. The MC1R is involved in the regulation of skin pigmentation [2,3]. The MC2R, implicated in steroidogenesis [3], is only activated by the adrenocorticotropic hormone (ACTH) and not other endogenous melanocortin ligands [10]. The MC3R and MC4R have been demonstrated to regulate appetite and energy homeostasis [4,5,6,11,12,13,14]. While the function of the MC5R has not been clearly elucidated in humans, this receptor has been linked to exocrine gland function in mice [1,7,8,15]. The melanocortin receptors are stimulated by endogenous agonists derived from the proopiomelanocortin (POMC) gene transcript [16], and include the α-, β-, and γ-melanocortin stimulating hormones (MSH) and ACTH, as previously reviewed [17,18]. Common to the endogenous agonists is a His-Phe-Arg-Trp tetrapeptide sequence, the minimum sequence when the N-terminal is acetylated and the C-terminal is amidated to produce a functional response in the frog (*Rana pipiens*) and lizard (*Anolis carolinensis*) skin bioassays [19,20]. The melanocortin system also contains two naturally occurring antagonists, agouti-signaling protein (ASP) and agouti-related protein (AGRP), which possess an Arg-Phe-Phe tripeptide motif hypothesized to be important for antagonist activity [21,22].

Studies in mice have indicated the important roles of the MC4R and MC3R in maintaining energy homeostasis. Knock-out (KO) MC4R mice are hyperphagic and obese compared to wildtype littermates [13]. While the MC3R may play a subtle role in regulating food intake [23], MC3R KO mice exhibit increased fat mass, reduced lean mass, and maintain a similar body weight compared to wildtype littermates [11,12]. Double MC3R/MC4R KO mice are significantly heavier than MC4R KO mice, suggesting non-redundant roles for the MC3R and MC4R in energy homeostasis [12,24,25,26]. Central administration of non-selective melanocortin agonists has been shown to decrease food intake in rodents [14,23,27], while the administration of MC3R/MC4R antagonists increases food intake [14,23,28]. Targeting the MC3R and MC4R may therefore lead to the development of treatments for metabolic disorders such as obesity, anorexia, and cachexia. Similar to MC4R KO mice, select human MC4R single nucleotide polymorphisms result in a hyperphagic and increased weight phenotype, as previously reviewed [29]. MC4R-selective ligands have been reported to reduce body weight, although these compounds possess side effects including increased blood pressure [30], erectile activity [31,32,33], and skin darkening [34,35]. While the skin darkening is most likely due to the stimulation of the MC1R, the increases in blood pressure [36] and erectile activity [37,38] are postulated to be MC4-mediated. In the case of blood pressure, the lack of reported adverse cardiovascular side effects of the MC4R-selective setmelanotide [34] indicates that this may be ligand-dependent. Though polymorphisms in the MC3R may predispose an individual to obesity, the role of the MC3R has not been clearly elucidated [39]. While selective probes and therapeutic compounds have been developed for the MC4R, there remains a need for MC3R-selective compounds to clarify the role of this receptor in energy homeostasis and as potential lead ligands in the development of novel weight management therapeutics that bypass the reported side effects of MC4R-selective ligands.

To identify novel scaffolds with agonist selectivity for the MC3R over the MC4R, our laboratory performed a tetrapeptide mixture-based positional scan [40]. From this study, a new scaffold tetrapeptide (Ac-His-Arg-(*p*I)DPhe-Tic-NH_2_) was identified that possessed nanomolar agonist potency at the MC3R (EC_50_ = 40 nM) and was an antagonist at the MC4R (pA_2_ = 7.0) [40]. Compared to the endogenous tetrapeptide melanocortin sequence (His-Phe-Arg-Trp), the new scaffold switched the Phe and Arg positions and incorporated a Tic residue in place of the Trp. A follow-up study utilized the most potent MC3R substitutions at each position within the tetrapeptide from the mixture-based positional scan, retaining the switched Phe and Arg positions (Arg or Gln were utilized in the second position, while (*p*I)DPhe or (*p*Cl)DPhe were substituted at the third position) [41]. A 100-fold selective MC3R versus MC4R agonist compound was identified (Ac-Val-Gln-(*p*I)DPhe-DTic-NH_2_) that did not possess antagonist potency at the MC4R and only partially stimulated the MC4R (less than 50% efficacy of NDP-MSH) [41]. Switching the Arg and Phe positions within the melanocortin tetrapeptide sequence may therefore lead to MC3R-selective ligands that may be further developed into probe and therapeutic lead compounds.

In previous studies examining the traditional melanocortin tetrapeptide sequence, the substitution of (*p*I)DPhe for Phe, yielding the ligand Ac-His-(*p*I)DPhe-Arg-Trp-NH_2_, resulted in a full agonist at the MC4R and a partial agonist with antagonist activity at the MC3R [42,43]. This contrasts to the observed MC3R agonism and partial agonism with antagonist activity at the MC4R for the scaffold Ac-His-Arg-(*p*I)DPhe-Tic-NH_2_, where switching to the Arg-(*p*I)DPhe motif and substituting Tic results in opposite MC3R–MC4R activities. Further examination of the DPhe *para*-position within the Ac-His-(*p*I)DPhe-Arg-Trp-NH_2_ scaffold demonstrated that this position influenced the efficacy at the MC3R. Full MC3R agonist activity was observed when DPhe, DTyr, (*p*Me)DPhe, (*p*CN)DPhe, (*p*F)DPhe, and (*p*Cl)DPhe were incorporated, while (*p*I)DPhe, (*p*Br)DPhe, (*p*CF_3_)DPhe, and (3,4-diCl)DPhe resulted in up to 50% receptor activation and micromolar to sub-micromolar antagonist potencies at the MC3R [42]. All of these substitutions maintained full MC4R efficacy [42].

**Figure 1:**
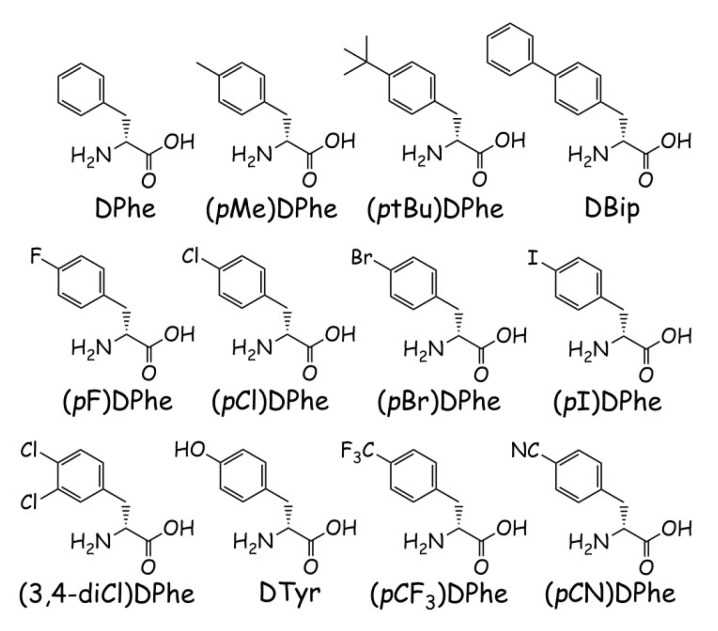
Structures and abbreviations of the amino acids used to replace the third amino acid in the peptide template Ac-His-Arg-Xxx-Tic-NH_2_.

Since switching the Phe and Arg positions results in MC3R agonism and MC4R partial agonism/antagonism for the Ac-His-Arg-(*p*I)DPhe-Tic-NH_2_ scaffold, it was hypothesized that further substitutions at the *para*-position might result in decreasing MC4R efficacy while retaining MC3R agonism. Therefore, a library of 12 peptides was synthesized based upon the scaffold Ac-His-Arg-(*p*X)DPhe-Tic-NH_2_ (substitutions for (*p*X)DPhe can be found in Figure 1) and assayed at the mouse MC1R, MC3R, MC4R, and MC5R, in order to understand how the *para*-position within this scaffold influences melanocortin receptor selectivity, potency, and efficacy.

## 2. Results

### Peptide Synthesis and Pharmacological Evaluation

Peptides were synthesized manually with microwave irradiation using standard Fmoc synthesis techniques [44,45] and purified using semi-preparative reverse-phase high-pressure liquid-chromatography (RP-HPLC). Peptide molecular mass was confirmed through ESI-MS (University of Minnesota Mass Spectrometry Laboratory), and each peptide was assessed for purity (>95%) using analytical RP-HPLC in two different solvent systems (acetonitrile and methanol; Table 1). Agonist pharmacology was measured at the mMC3R, mMC4R, and mMC5R using a colorimetric β-galactosidase assay that measures cAMP production [46]. Agonist pharmacology was assessed at the mMC1R using the Amplified Luminescent Proximity Homogenous Assay Screen (AlphaScreen, PerkinElmer), as previously described [47,48,49]. The MC2R is only stimulated by ACTH, and was not examined in this study. For both assays, HEK293 cells stably expressing the mMCRs were used. For agonist assays, the peptide ligands NDP-MSH [50] and Ac-His-DPhe-Arg-Trp-NH_2_ [51] were used as positive controls. Ligands were considered full agonists if they stimulated the receptor to >90% of the maximal signal of NDP-MSH and were considered inactive if they did not stimulate the receptor to at least 20% of the signal of NDP-MSH at a 100 µM concentration. Compounds that did not possess at least 50% of the maximal NDP-MSH signal were assessed for antagonist pharmacology using a Schild assay design [52], with NDP-MSH as the agonist. Compounds that were within 3-fold potency range were considered equipotent and within the inherent experimental error of the assays.

**Figure 2:**
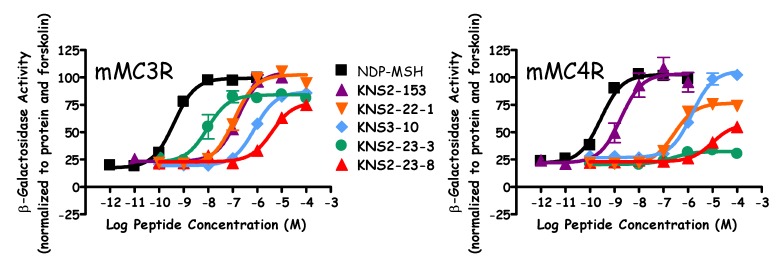
Illustration of the agonist pharmacology of NDP-MSH, **KNS2-153**, **KNS2-22-1**, **KNS3-10**, **KNS2-23-3**, and **KNS2-23-8** at the mMC3R and mMC4R.

**Figure 3:**
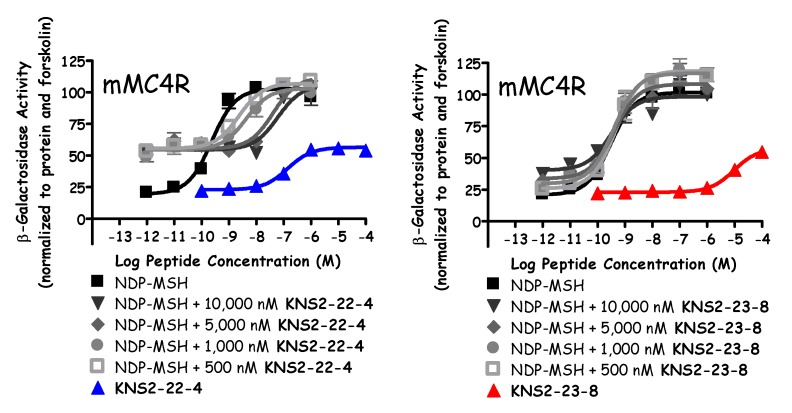
Illustration of the antagonist pharmacology of **KNS2-22-4** and **KNS2-23-8** at the mMC4R.

Similar to prior reports [49,51], the Ac-His-DPhe-Arg-Trp-NH_2_ peptide (**KNS2-153**) possessed agonist potencies of 10, 190, 12, and 5 nM at the mMC1R, mMC3R, mMC4R, and mMC5R, respectively (Figure 2, Table 2 and Table 3). The lead ligand for the current series (**KNS2-22-4**) switched the Arg and DPhe positions, utilized a (*p*I)DPhe in the place of DPhe, and substituted a Tic residue in the place of Trp compared to **KNS2-153**. These alterations resulted in a ligand that maintained full agonist efficacy at the mMC1R, mMC3R, and mMC5R (EC_50_ = 0.7, 13, and 5 nM, respectively; Table 2 and Table 3), and partial agonist efficacy at the mMC4R (40% of the NDP-MSH signal, EC_50_ = 150 nM; Figure 3). An antagonist pA_2_ value of 7.3 was observed for **KNS2-22-4** at the mMC4R (Figure 3). In prior studies, this compound was observed to possess nanomolar agonist potency at the MC3R (30–40 nM), partial agonist stimulation of the MC4R, and sub-micromolar antagonist potency at the MC4R (pA_2_ of 6.6–7) [40,41], similar to the results in the present study.

Replacing (*p*I)DPhe with (*p*Br)DPhe (**KNS2-22-3**) resulted in similar potencies at the melanocortin receptors assayed compared to **KNS2-22-4**, although higher efficacy was observed at the mMC4R (55% maximal NDP-MSH signal). The (*p*Cl)DPhe-substituted **KNS2-22-1** maintained similar potencies compared to **KNS2-22-4** at the mMC1R and mMC5R, but was 9-fold less potent at the mMC3R (120 nM, Figure 2) and possessed an increased partial agonist response relative to NDP-MSH (70%, EC_50_ = 280 nM) at the mMC4R (Figure 2) compared to **KNS2-22-4**. The Ac-His-Arg-(*p*Cl)DPhe-Tic-NH_2_ (**KNS2-22-1**) tetrapeptide was previously reported to possess agonist pharmacology at the mMC3R (110 nM) and partial agonist activity at the mMC4R (EC_50_ = 140 nM), similar to the values observed in the present study [41]. While similar potency relative to **KNS2-22-4** was observed for the (*p*F)DPhe-substituted **KNS2-22-2** at the mMC1R, this substitution decreased potency at the mMC3R and mMC5R (30- and 14-fold as compared to **KNS2-22-4**, respectively). Similar to **KNS2-22-1**, a 70% partial agonist response at the mMC4R (EC_50_ = 560 nM) was observed for **KNS2-22-2**.

The DPhe-substituted **KNS3-10** possessed decreased agonist potency compared to **KNS2-22-4** at the mMC1R (6-fold), mMC3R (70-fold), and mMC5R (5-fold). This substitution resulted in a partial agonist response at the mMC3R and mMC5R (85% and 65% maximal NDP-MSH signal, respectively), and was the only compound in the series to possess full agonist efficacy at the mMC4R (EC_50_ = 3 µM; Figure 2). The only di-substituted ring examined, (3,4-diCl)DPhe (**KNS2-23-4**), possessed decreased potency at the mMC1R (7-fold), mMC3R (30-fold), and mMC5R (14-fold) compared to **KNS2-22-4**, and did not result in stimulation of the mMC4R at concentrations up to 100 μM. This substitution resulted in micromolar antagonist potency at the mMC4R (pA_2_ = 6.2).

Replacing (*p*I)DPhe with (*p*Me)DPhe (**KNS2-23-7**) resulted in similar agonist potencies at the mMC1R and mMC5R compared to **KNS2-22-4**, an 8-fold decreased potency at the mMC3R, and stimulated the mMC4R up to 50% of the maximal NDP-MSH signal (EC_50_ = 700 nM). Substituting a *p*-trifluoromethyl group (**KNS2-23-6**) retained similar potency at the mMC5R compared to **KNS2-22-4**, but decreased potency at the mMC1R and mMC3R (7-fold for both). This substitution resulted in 20% stimulation of the mMC4R (relative to NDP-MSH), with an agonist potency of 600 nM and an antagonist pA_2_ value of 6.5. The incorporation of (*p*tBu)DPhe resulted in tetrapeptide **KNS2-23-3**, with decreased potency at the mMC1R (13-fold) compared to **KNS2-22-4**, similar potency at the mMC3R and mMC5R, and produced a partial agonist response (85% relative to NDP-MSH) at the mMC3R. This substitution resulted in minimal agonist activity (<20%) at the mMC4R (Figure 2), but resulted in the second highest antagonist potency observed at the mMC4R (pA_2_ = 6.8). When another aromatic ring was extended from the *para*-position (DBip, **KNS2-23-1**), similar potencies at the mMC1R, mMC3R, and mMC5R were observed compared to **KNS2-22-4**, with decreased agonist (1.4 µM) and antagonist (pA_2_ = 5.9) potencies at the mMC4R.

The least potent compounds possessed a hydroxyl (**KNS2-23-9**) or nitrile (**KNS2-23-8**) group at the *para*-position. The substitution of DTyr (**KNS2-23-9**) resulted in potencies of 40 nM, 4300 nM, and 1000 nM at the mMC1R, mMC3R, and mMC5R, respectively, and did not possess agonist or antagonist activity at the mMC4R in the concentrations assayed. An 85% partial agonist response was observed at the mMC3R. Similar agonist potencies of 27 nM, 4000 nM, and 500 nM at the mMC1R, mMC3R, and mMC5R were observed for **KNS2-23-8**, with partial efficacy at the mMC3R (75%). At 100 μM concentrations, this ligand was able to partially stimulate the mMC4R (40% of the maximal NDP-MSH signal; Figure 2 and Figure 3) and did not result in antagonist activity (Figure 3).

## 3. Discussion

Previous results exploring the DPhe *para*-position in the Ac-His-DPhe-Arg-Trp-NH_2_ scaffold resulted in full MC4R agonists with different MC3R agonist and antagonist activities [42]. Select substitutions resulted in full MC3R agonist efficacy, while others resulted in partial receptor activation at 100 μM concentrations and micromolar to sub-micromolar antagonist potencies [42]. Thus, a DPhe-Arg motif resulted in full agonism at the MC4R and full to partial agonism at the MC3R accompanied by antagonist activity (dependent on the DPhe *para*-position). Due to the MC3R agonism and MC4R antagonism observed in the Ac-His-Arg-(*p*I)DPhe-Tic-NH_2_ ligand [40,41], it was hypothesized that different substitutions at the DPhe *para*-position within this scaffold (possessing an Arg-DPhe motif and a Tic residue in position 4) may modulate MC4R agonist efficacy. The results in Table 3 demonstrate that the efficacy at the mMC4R was modulated by various *para*-substitutions. Full agonism was observed for the ligand Ac-His-Arg-DPhe-Tic-NH_2_ (**KNS3-10**) at the mMC4R (Figure 2), and an additional four substitutions resulted in over 50% agonist efficacy at the mMC4R ((*p*Br)DPhe, (*p*Cl)DPhe, (*p*F)DPhe, and (*p*Me)DPhe). Modest agonist efficacy (20–50%) was observed for four ligands (possessing the (*p*I)DPhe, (*p*CF_3_)DPhe, DBip, and (*p*CN)DPhe substitutions), and three substitutions ((3,4-diCl)DPhe, (*p*tBu)DPhe, and DTyr) resulted in compounds that did not produce >20% response of the maximal NDP-MSH signal at up to 100 μM concentrations at the mMC4R. A partial agonist response at the mMC3R was also observed for four of the ligands. Thus, the *para*-substitution at the DPhe position within the Ac-His-Arg-(*p*I)DPhe-Tic-NH_2_ scaffold modulates agonist efficacy at both the mMC3R and mMC4R, with the Arg-DPhe motif in general resulting in a more efficacious response at the mMC3R.

Several compounds from this study may be useful lead ligands in the development of MC3R/MC4R-selective compounds. One compound (**KNS2-23-9**) possessed micromolar mMC3R agonist potency and did not possess agonist or antagonist activity at the mMC4R. An additional three compounds were at least 100-fold selective agonists for the mMC3R over the mMC4R (**KNS2-23-4**, **KNS2-23-3**, and **KNS2-23-1**), though these three ligands possessed micromolar to sub-micromolar mMC4R antagonist potencies. Further optimization to increase MC3R potency and efficacy, and to minimize MC4R pharmacology, may be required to develop selective MC3R ligands that can elucidate the roles of the MC3R. The use of MC3R KO and MC4R KO mice may also be used with the present ligands to begin to clarify the roles of the different melanocortin receptors in vivo. Alternatively, three compounds (**KNS2-22-4**, **KNS2-23-6**, and **KNS2-23-3**) possessed mMC3R agonist potencies of less than 100 nM and were sub-micromolar potent mMC4R antagonists. Further optimization of this dual pharmacology (increased MC3R agonism with increased MC4R antagonism) might result in novel tool compounds that can characterize the in vivo role of the MC3R and MC4R in the regulation of food intake.

While these substitutions had an effect on efficacy at the mMC3R and mMC4R, all compounds assayed were full agonists at the mMC1R, and only one compound was not a full agonist at the mMC5R (**KNS3-10**, stimulating the mMC5R to 65% of the maximal NDP-MSH response). It therefore appears that the Arg-DPhe position switch may only lead to mMC3R over mMC4R selectivity. Potency trends at the mMC1R and mMC5R were similar to that at the mMC3R. The two compounds that were micromolar potent mMC3R agonists (**KNS2-23-9** and **KNS2-23-8**) were also the least potent mMC1R (40 and 27 nM, respectively) and mMC5R (1000 and 500 nM, respectively) agonists. While no compound was significantly more potent than the Ac-His-DPhe-Arg-Trp-NH_2_ ligand at the mMC5R, five ligands resulted in at least a 10-fold potency increase at the mMC1R (**KNS2-22-4**, **KNS2-22-3**, **KNS2-22-1**, **KNS2-23-7**, and **KNS2-23-1**).

Another report investigated the *para*-position within the Ac-His-DPhe-Arg-Trp-NH_2_ scaffold for MC1R selectivity [53]. In addition to *p*F, *p*Cl, *p*Br, and *p*CF_3_ substitutions, Arg was replaced with a neutral Nle residue due to hypothesized interactions with the Arg and basic residues in the MC3R and MC4R [53]. As a general trend, these substitutions increased binding affinity at the MC1R compared to the other melanocortin receptors, as well as increased agonist selectivity for the MC1R [53]. Intraperitoneal (i.p.) injection of the *p*CF_3_ substituted ligand resulted in in vivo pigmentation effects when administered to *Anolis carolinesis* lizards [53]. Our results indicate that switching the Phe-Arg positions and substituting Tic for Trp may also increase MC1R potency. When combined with the Nle substitution at the Arg position, these substitution patterns may result in increased MC1R selectivity and/or potency.

## 4. Materials and Methods

### 4.1. Reagents

4-(2′,4′-Dimethoxyphenyl-Fmoc-aminomethyl)phenoxyacetyl-MBHA resin (rink-amide-MBHA (100–200 mesh), 0.66 equivalents/g substitution), 2-(1H-benzotriazol-1-yl)1,1,3,3,-tetramethyluronium hexafluorophosphate (HBTU), and the amino acids Fmoc-His(Trt), Fmoc-Arg(Pbf), Fmoc-DPhe, Fmoc-Trp(Boc), Fmoc-(*p*F)DPhe, and Fmoc-(*p*Cl)DPhe were purchased from Peptides International (Louisville, KY, USA). Fmoc-(*p*Br)DPhe, Fmoc-(3,4-diCl)DPhe, Fmoc-(*p*CN)DPhe, Fmoc-(*p*Me)DPhe, and Fmoc-(*p*tBu)DPhe were purchased from BACHEM (San Carlos, CA, USA). Fmoc-(*p*I)DPhe was purchased from Alfa Aesar (Tewksbury, MA, USA). Fmoc-(*p*CF_3_)DPhe was purchased from Chem Cruz (Dallas, TX, USA). Fmoc-d-4,4′-biphenylalanine (Fmoc-Bip) and Fmoc-1,2,3,4-tetrahydroisoquinoline-3-carboxylic acid (Fmoc-Tic) were purchased from Synth Tech (Albony, OR, USA). Fmoc-DTyr(But) was acquired from Advanced Chemtech (Louisville, KY, USA). Triisopropylsilane (TIS), dimethyl sulfoxide (DMSO), *N,N*-diisopropylethylamine (DIEA), 1,2-ethanedithiol (EDT), piperidine, pyridine, and trifluoroacetic acid (TFA) were purchased from Sigma-Aldrich (St. Louis, MO, USA). Acetonitrile (MeCN), *N,N-*dimethylformamide (DMF), dichloromethane (DCM), methanol (MeOH), and acetic anhydride were purchased from Fisher Scientific. All reagents were ACS grade or higher and were used without further purification.

### 4.2. Peptide Synthesis

Peptides were synthesized on a CEM Discover SPS manual microwave synthesizer using standard fluorenyl-9-methyloxycarbonyl (Fmoc) methodology [44,45]. The rink-amide resin was added to a fritted polypropylene reaction vessel (25 mL CEM reaction vessel). The resin was allowed to swell in DCM for 1 h. Deprotection of the Fmoc group consisted of two steps: (1) 20% piperidine in DMF at rt for two minutes, followed by (2) 20% piperidine in DMF using microwave irradiation for 4 min at 75 °C with 30 W. The resin was washed with DMF, and the presence of a free amine was assessed using the ninhydrin [54] or chloranil [55] (for the Tic residue) tests. Coupling reactions were carried out with 3.1 equivalents (eq) of the incoming Fmoc-protected amino acid, 3 eq HBTU, and 5 eq DIEA using microwave irradiation (5 min, 75 °C, 30 W). A lower temperature (50 °C) was utilized for His. For Arg coupling, higher equivalents of Arg (5.1 eq), HBTU (5 eq), and DIEA (7 eq) were used with a longer (10 min) microwave irradiation time. Following resin washing with DMF, the completeness of the coupling reactions was assessed with the ninhydrin or chloranil tests, and amino acids were recoupled if necessary. Following the coupling of the N-terminal His residue, the final Fmoc group was removed and the N-terminal was acetylated with 3:1 acetic anhydride:pyridine for 30 min at rt. Peptides were side-chain deprotected and cleaved from the resin for 2 h using a 91:3:3:3 TFA:thioanisole:TIS:H_2_O solution, except for **KNS2-23-9** (Ac-His-Arg-DTyr-Tic-NH_2_), which was cleaved in a 91:3:3:3 TFA:EDT:TIS:H_2_O solution. After cleavage, peptides were precipitated in ice-cold diethyl ether, and pelleted using a Sorvall Legend XTR centrifuge using a swinging bucket rotor (4000 rpm for 4 min at 4 °C). The peptide was washed with diethyl ether and pelleted at least three times before drying overnight in a desiccator.

The peptides were purified by RP-HPLC on a semipreparative C18 reverse-phase column (Vydac 2181010, 10 × 250 mm) using a Shimadzu UV detector (Shimadzu, Kyoto, Japan). The collected fractions were concentrated on a rotary evaporator and lyophilized. The purified compounds were characterized analytically by RP-HPLC on an analytical C18 reverse-phase column (Vydac 218104; Hichrom, Theale, UK) using two solvent systems—methanol and acetonitrile. Peptides were determined to be greater than 95% pure as assessed by peak area at 214 nm, and the correct average molecular mass was confirmed using ESI/TOF-MS (Bruker, BioTOF II, University of Minnesota Department of Chemistry Mass Spectrometry Laboratory, Minneapolis, MN, USA).

### 4.3. AlphaScreen Bioassay

Peptide ligands were dissolved in DMSO at stock concentrations of 10^−2^ M. To assess the pharmacological activity of the tetrapeptides at the mMC1R, HEK293 cells stably expressing the mMC1R were stimulated with the ligands using the cAMP AlphaScreen assay (PerkinElmer) according to the manufacturer’s instruction and as previously described [47,49,56].

Cells were grown at 37 °C with 5% CO_2_ in cell media (Dulbecco’s Modified Eagle’s Medium (DMEM) containing 10% newborn calf serum (NCS) and 1% penicillin-streptomycin) in 10 cm plates to 70–95% confluency. Cells were dislodged with Versene (Gibco) at 37 °C, and 10,000 cells/well were plated in a 384-well plate (Optiplate) with freshly made stimulation buffer (Hank’s Balanced Salt Solution (HBSS, 1×), 0.5 mM 3-isobutyl-1-methylxanthine (IBMX), 5 mM HEPES, and 0.1% bovine serum albumin (BSA), pH = 7.4) with 0.5 μg anti-cAMP acceptor beads per well. The cells were stimulated with the addition of 5 μL stimulation buffer containing peptide (a seven-point dose response curve was used starting with peptide concentrations of 10^−4^ to 10^−7^ M, determined by ligand potency) or forskolin (10^−4^ M) and incubated in the dark at rt for 2 h.

Following stimulation, biotinylated cAMP (0.62 μM) and streptavidin-coated donor beads (0.5 μg) were added to the wells in a subdued light environment with 10 μL lysis buffer (0.3% Tween-20, 5 mM HEPES, and 0.1% BSA, pH = 7.4). Plates were incubated for an additional 2 h in the dark. Post incubation, the plates were read by an EnSpire plate reader (PerkinElmer, Waltham, MA, USA).

### 4.4. β-Galactosidase Assay

The peptide ligands were assessed for pharmacological activity at the mMC3R, mMC4R, and mMC5R using a β-galactosidase assay. Briefly, HEK293 cells stably expressing the MC3R, MC4R, or MC5R were plated into a 10 cm dish and grown to 40% confluency. The HEK293 cells were transfected with 4 µg of CRE/β-galactosidase using the calcium-phosphate method, as previously described [46]. Cells (5000 to 15,000) were plated on collagen-treated Nunclon Delta Surface 96-well plates (Thermo Fisher Scientific) and incubated at 37 °C with 5% CO_2_. Plates were stimulated 48 h post-transfection with 100 μL solutions of peptide (a seven-point dose response curve with concentrations between 10^−4^ to 10^−12^ M, depending on potency) or forskolin (10^−4^ M) in assay media (DMEM containing 0.1 mg/mL BSA and 0.1 mM IBMX) for 6 h. The assay media was aspirated and 50 µL of lysis buffer (250 mM Tris-HCl, 0.1% Triton X-100, pH 8.0) was added to each well. Plates were stored at −80 °C for up to two weeks.

Thawed plates were assessed for protein content and assayed for β-galactosidase activity. Relative protein concentration was determined by adding 10 µL of cell lysate to 200 µL of a 1:5 dilution of Bio Rad G250 protein dye in a 96-well plate. Absorbance was measured using a 96-well plate reader (Molecular Devices) at λ = 595 nm. To determine β-galactosidase activity, 40 µL of 0.5% BSA in phosphate buffered saline (PBS) (37 °C) and 150 µL of the β-galactosidase substrate (60 mM Na_2_HPO_4_, 1 mM MgCl_2,_ 10 mM KCl, 50 mM 2-mercaptoethanol, and 660 µM 2-nitrophenyl β-d-galactosidase) were added to the remaining 40 µL of cell lysate. Plates were incubated at 37 °C and periodically read on the 96-well plate reader until the absorbance at λ = 405 nm reached approximately 1.0 relative absorbance units for the positive controls.

### 4.5. Data Analysis

The EC_50_ and pA_2_ values represent the mean of duplicate replicates performed in at least three independent experiments. The EC_50_ and pA_2_ values and their associated standard errors (SEM) were determined by fitting the data to a nonlinear least-squares analysis using the PRISM program (v4.0, GraphPad Inc., San Diego, CA, USA). The ligands were assayed as TFA salt and not corrected for peptide content.

## 5. Conclusions

The tetrapeptide Ac-His-Arg-(*p*I)DPhe-Tic-NH_2_, possessing a switched Arg-DPhe motif and Tic at the fourth position relative to the Ac-His-DPhe-Arg-Trp-NH_2_ melanocortin agonist sequence, was characterized to be an MC3R agonist/MC4R antagonist ligand following a mixture-based positional scan to identify MC3R agonist-selective ligands. Previous characterization of the DPhe *para*-position within the Ac-His-DPhe-Arg-Trp-NH_2_ scaffold indicated that substitutions influenced MC3R efficacy while maintaining full MC4R agonism. It was therefore hypothesized that different substitutions at the DPhe *para-*position in the Ac-His-Arg-(*p*I)DPhe-Tic-NH_2_ scaffold might modulate MC4R efficacy while maintaining MC3R agonism. A range of MC4R efficacies was observed from the library of 12 compounds, including one full agonist and three ligands that possessed no agonist activity at concentrations up to 100 μM. Efficacy at the MC3R was also modulated, though all compounds maintained at least at 75% stimulation of the MC3R relative to NDP-MSH. Thus, the inversion of the Arg and DPhe positions within the melanocortin tetrapeptide sequences appears to result in preferential MC3R agonism over MC4R, a useful design motif for the development of MC3R-selective ligands that may serve as novel probe and lead ligands in the treatment of various disorders of altered energy balance.

## Figures and Tables

**Table 1:** Analytical data for peptides synthesized in this study ^a^.

Peptide	Sequence	Retention Time (min)		M (Calculated)	M + H (Observed)	Purity (%)
		System 1	System 2			
**KNS2-153**	Ac-His-DPhe-Arg-Trp-NH_2_	10.1	15.6	685.3	686.4	>98
**KNS2-22-4**	Ac-His-Arg-**(*p*I)DPhe**-Tic-NH_2_	14.9	23.6	784.2	785.3	>97
**KNS2-22-3**	Ac-His-Arg-**(*p*Br)DPhe**-Tic-NH_2_	14.9	23.2	736.3, 738.3 ^b^	737.3, 739.3 ^b^	>98
**KNS2-22-1**	Ac-His-Arg-**(*p*Cl)DPhe**-Tic-NH_2_	14.6	22.7	692.3	693.5	>97
**KNS2-22-2**	Ac-His-Arg-**(*p*F)DPhe**-Tic-NH_2_	13.5	20.8	676.3	677.5	>95
**KNS3-10**	Ac-His-Arg-**DPhe**-Tic-NH_2_	12.8	20.1	658.3	659.5	>99
**KNS2-23-4**	Ac-His-Arg-**(3,4-diCl)DPhe**-Tic-NH_2_	15.6	24.2	726.3	727.4	>97
**KNS2-23-7**	Ac-His-Arg-**(*p*Me)DPhe**-Tic-NH_2_	14.3	22.3	672.4	673.5	>97
**KNS2-23-6**	Ac-His-Arg-**(*p*CF_3_)DPhe**-Tic-NH_2_	15.0	23.4	726.7	727.5	>98
**KNS2-23-3**	Ac-His-Arg-**(*p*tBu)DPhe**-Tic-NH_2_	17.5	26.5	714.4	715.4	>95
**KNS2-23-1**	Ac-His-Arg-**DBip**-Tic-NH_2_	16.7	25.9	734.3	735.5	>96
**KNS2-23-9**	Ac-His-Arg-**DTyr**-Tic-NH_2_	10.5	15.0	674.3	675.4	>96
**KNS2-23-8**	Ac-His-Arg-**(*p*CN)DPhe**-Tic-NH_2_	11.5	17.2	683.3	684.3	>97

^a^ HPLC retention time (min) for peptides in solvent system 1 (10% acetonitrile in 0.1% trifluoroacetic acid/water and a gradient to 90% acetonitrile over 35 min) or solvent system 2 (10% methanol in 0.1% trifluoroacetic acid/water and a gradient to 90% methanol over 35 min). An analytical Vydac C18 column (Vydac 218TP104) was used with a flow rate of 1.5 mL/min. The peptide purity was determined by HPLC at a wavelength of 214 nm. ^b^ Two peaks were observed for the (*p*Br)DPhe amino acid due to the approximately equal natural abundance of ^79^Br and ^81^Br.

**Table 2:** Tetrapeptide pharmacology at the mouse melanocortin-1 receptor using the AlphaScreen cyclic adenosine monophosphate (cAMP) assay ^a^.

Peptide	Sequence	mMC1R EC_50_ (nM)
NDP-MSH	Ac-Ser-Tyr-Ser-Nle-Glu-His-DPhe-Arg-Trp-Gly-Lys-Pro-Val-NH_2_	0.009 ± 0.002
**KNS2-153**	Ac-His-DPhe-Arg-Trp-NH_2_	10 ± 3
**KNS2-22-4**	Ac-His-Arg-**(*p*I)DPhe**-Tic-NH_2_	0.7 ± 0.2
**KNS2-22-3**	Ac-His-Arg-**(*p*Br)DPhe**-Tic-NH_2_	0.7 ± 0.3
**KNS2-22-1**	Ac-His-Arg-**(*p*Cl)DPhe**-Tic-NH_2_	0.8 ± 0.2
**KNS2-22-2**	Ac-His-Arg-**(*p*F)DPhe**-Tic-NH_2_	1.8 ± 0.7
**KNS3-10**	Ac-His-Arg-**DPhe**-Tic-NH_2_	4.6 ± 0.4
**KNS2-23-4**	Ac-His-Arg-**(3,4-diCl)DPhe**-Tic-NH_2_	5 ± 2
**KNS2-23-7**	Ac-His-Arg-**(*p*Me)DPhe**-Tic-NH_2_	1.0 ± 0.3
**KNS2-23-6**	Ac-His-Arg-**(*p*CF_3_)DPhe**-Tic-NH_2_	5 ± 1
**KNS2-23-3**	Ac-His-Arg-**(*p*tBu)DPhe**-Tic-NH_2_	9 ± 3
**KNS2-23-1**	Ac-His-Arg-**DBip**-Tic-NH_2_	0.6 ± 0.1
**KNS2-23-9**	Ac-His-Arg-**DTyr**-Tic-NH_2_	40 ± 10
**KNS-2-23-8**	Ac-His-Arg-(*p*CN)DPhe-Tic-NH_2_	27 ± 6

^a^ The indicated error represents the standard error of the mean determined from at least three independent experiments performed in duplicate replicates.

**Table 3:** Tetrapeptide pharmacology at the mouse melanocortin-3, -4, and -5 receptors using the β-Galactosidase cAMP assay ^a^.

Peptide	Sequence	mMC3R EC_50_ (nM)	mMC4R		mMC5R EC_50_ (nM)
			EC_50_ (nM)	pA_2_	
**NDP-MSH**	Ac-Ser-Tyr-Ser-Nle-Glu-His-DPhe-Arg-Trp-Gly-Lys-Pro-Val-NH_2_	0.52 ± 0.05	0.32 ± 0.02	-	3.4 ± 0.7
**KNS2-153**	Ac-His-DPhe-Arg-Trp-NH_2_	190 ± 40	12 ± 3	-	5 ± 2
**KNS2-22-4**	Ac-His-Arg-**(*p*I)DPhe**-Tic-NH_2_	13 ± 2	Partial Agonist 150 ± 40 (40% NDP)	7.3 ± 0.8	5 ± 1
**KNS2-22-3**	Ac-His-Arg-**(*p*Br)DPhe**-Tic-NH_2_	90 ± 20	Partial Agonist 290 ± 50 (55% NDP)	-	9.7 ± 0.5
**KNS2-22-1**	Ac-His-Arg-**(*p*Cl)DPhe**-Tic-NH_2_	120 ± 20	Partial Agonist 280 ± 60 (70% NDP)	-	18 ± 6
**KNS2-22-2**	Ac-His-Arg-**(*p*F)DPhe**-Tic-NH_2_	450 ± 70	Partial Agonist 560 ± 60 (70% NDP)	-	70 ± 40
**KNS3-10**	Ac-His-Arg-**DPhe**-Tic-NH_2_	Partial Agonist 900 ± 200 (85% NDP)	3000 ± 2000	-	Partial Agonist 200 ± 30 (65% NDP)
**KNS2-23-4**	Ac-His-Arg-**(3,4-diCl)DPhe**-Tic-NH_2_	400 ± 100	>100,000	6.15 ± 0.05	70 ± 7
**KNS2-23-7**	Ac-His-Arg-**(*p*Me)DPhe**-Tic-NH_2_	110 ± 20	Partial Agonist 700 ± 200 (50% NDP)	-	17 ± 4
**KNS2-23-6**	Ac-His-Arg-**(*p*CF_3_)DPhe**-Tic-NH_2_	90 ± 30	Partial Agonist 600 ± 300 (20% NDP)	6.5 ± 0.2	13 ± 4
**KNS2-23-3**	Ac-His-Arg-**(*p*tBu)DPhe**-Tic-NH_2_	Partial Agonist 13 ± 4 (85% NDP)	>100,000	6.8 ± 0.3	3.4 ± 0.3
**KNS2-23-1**	Ac-His-Arg-**DBip**-Tic-NH_2_	14 ± 2	Partial Agonist 1400 ± 700 (45% NDP)	5.9 ± 0.2	7.6 ± 0.7
**KNS2-23-9**	Ac-His-Arg-**DTyr**-Tic-NH_2_	Partial Agonist 4200 ± 800 (85% NDP)	>100,000	<5.5	1000 ± 500
**KNS2-23-8**	Ac-His-Arg-**(*p*CN)DPhe**-Tic-NH_2_	Partial Agonist 4000 ± 1000 (75% NDP)	40% @ 100 µM	<5.5	500 ± 100

^a^ The indicated error represents the standard error of the mean determined from at least three experiments performed in duplicate replicates. The value of >100,000 nM indicates that the compound was assayed but no agonist activity was observed up to a concentration of 100 μM. A percentage denotes the percent maximal stimulatory response observed at 100 μM, but not enough stimulation was observed to determine an EC_50_ value. Partial agonist indicates a partial agonist with the percent maximal stimulation (relative to NDP-MSH) and the apparent EC_50_ value. Antagonist pA_2_ values were determined using a Schild analysis [52] and the agonist NDP-MSH. The value of <5.5 indicates that no antagonist potency was observed in the highest antagonist concentration range assayed (10,000, 5000, 1000, and 500 nM). A dash (-) indicates that the compound was not assayed as an antagonist at the mMC4R.
